# Attitudes and Preferences Towards Screening for Dementia From the Perspectives of Healthcare Professionals: An Updated Systematic Review

**DOI:** 10.1002/gps.70057

**Published:** 2025-02-20

**Authors:** Ríona Mc Ardle, Abigail Dsouza, Alexander Hagan, Amani Al‐Oraibi, Matilda Hanjari, Blossom C. M. Stephan, Carol Brayne, Louise Lafortune, Manpreet Bains, Nadeem Qureshi, Louise Robinson

**Affiliations:** ^1^ Translational and Clinical Research Institute Newcastle University Tyne UK; ^2^ Population Health Sciences Institute Newcastle University Tyne UK; ^3^ PRISM Research Group Lifespan and Population Health School of Medicine University of Nottingham Nottingham UK; ^4^ Department of Respiratory Sciences University of Leicester Leicester UK; ^5^ Development Centre for Population Health University of Leicester Leicester UK; ^6^ Faculty of Health and Life Sciences Institute for Allied Health Sciences Research De Montfort University Leicester UK; ^7^ Dementia Centre of Excellence EnAble Institute Curtin University Bentley Western Australia Australia; ^8^ Institute of Mental Health University of Nottingham Nottingham UK; ^9^ Cambridge Public Health University of Cambridge Forvie Site Robinson Way Cambridge UK

**Keywords:** Alzheimer's disease, attitudes, community health services, dementia, mental status and dementia tests, preferences, primary health care

## Abstract

**Objectives:**

Approximately 55 million people are living with dementia globally. Global policies have suggested that screening for dementia in asymptomatic populations may support risk‐reduction approaches to stem the rising numbers of people with the condition. A previous systematic review of literature up to 2012 indicated that healthcare professionals negatively view dementia screening; however, the research and clinical landscape has made significant advances in the last decade. Therefore, the aim of this systematic review is to re‐examine the attitudes and preferences of healthcare professionals since 2012 regarding primary and community care‐based dementia screening.

**Methods:**

This review was pre‐registered on PROSPERO (CRD42024531455) and followed PRISMA guidelines. Key terms relevant to the aim were input into six databases, and articles between 2012 and 2024 were considered. Titles, abstracts and full texts were independently screened by two reviewers. Articles were eligible for inclusion if peer‐reviewed, written in English, considered primary or community care‐based dementia screening and included healthcare perspectives from either quantitative or qualitative methods.

**Results:**

From 18,732 identified titles, 19 articles were included in this review. Seventeen studies presented perspectives from primary care practitioners. Key findings suggest that healthcare professionals have mixed views regarding their willingness to conduct dementia screening, although limited studies suggest an interest in engaging in dementia risk reduction. Common influences on perspectives included knowledge, skills and training; resource provision; access to a dedicated screening service and referral pathways; and stigma.

**Conclusions:**

These findings suggest that healthcare professionals' perspectives and resource are not aligned with international policies promoting dementia screening. When considering implementing evidence‐based dementia screening in the future, a dedicated screening service is recommended.

**Summary**

- Dementia screening to support primary and secondary prevention of the condition have been suggested by global policies.
- Implementation of screening programmes for any condition require acceptability amongst healthcare professionals.
- Findings from this review suggest primary and community‐care practitioners have mixed views regarding willingness to conduct dementia screening.
- Requirements for a future screening programme include increased resource, access to a dedicated service and referral pathway, and evidence of benefit.

## 1 Introduction

Dementia is the most common neurodegenerative condition to impact older adults, characterised by multiple cognitive deficits which impair functional abilities [1]. An estimated 55 million people are living with dementia globally, with numbers predicted to rise to 139 million by 2050 [3]; leading to the World Health Organisation (WHO) pronouncing dementia a global health priority [1]. However, dementia is not considered an inevitable part of ageing; up to 45% of all dementia cases potentially preventable [2]. A number of modifiable risk factors have been identified including lifestyle (e.g. physical, cognitive and social activities, smoking and excessive alcohol consumption) and healthcare factors (e.g. preventing and managing chronic conditions such as diabetes and hypertension, detecting hearing loss) [2, 3].

The 2023 World Alzheimer's Report stated that ‘in the absence of a cure or a treatment that is globally accessible, risk reduction remains the most feasible and proactive way to combat dementia’ [3]. In addition, research indicates that risk‐reduction strategies may slow progression of the dementia in those identified early in the disease course [3]. Screening for dementia in primary and community care is one approach to address dementia risk reduction in asymptomatic populations [4]. Additionally, dementia screening may support people to engage in clinical trials with access to potential treatments earlier, raise awareness of potential symptoms, enhance pathways to diagnosis and allow time for the individual and their families to make decisions about disease management and advanced care planning [5, 6]. Therefore, understanding the perspectives and attitudes of healthcare professionals is important when considering how we develop, validate and implement approaches for identifying dementia earlier especially at pre‐symptomatic stages. This is particularly relevant in light of the recent advances in blood, digital and artificial intelligence derived markers and positive results from randomised controlled trials of individualised interventions for dementia risk reduction (e.g. FINGERS) [6, 7, 8, 9, 10, 11, 12, 13].

In 2015, Martin, et al. [14] systematically reviewed the literature up to 2012 pertaining to healthcare professionals' views on dementia screening. Dementia screening was largely negatively perceived, with key barriers including lack of dementia awareness, belief in benefit, suitable screening tools, clinician time and appropriate treatments, and concerns regarding patients' health and co‐morbidities, cost of screening, impact of disclosure of results and stigma regarding dementia. However, the dementia policy and priority landscape has changed in the last decade. For example, although there has not yet been sufficient evidence for the benefits of population screening for dementia [15], results from a 2019 survey in the UK indicate that the public are generally willing to know their risk of developing dementia before symptoms occur [16]. Additionally, a recent systematic review exploring patient/public attitudes in under‐served groups (i.e. ethnic minorities and low socio‐economic groups) primarily in the USA between 2012 and 2023 indicated a high willingness to undergo dementia screening due to perceived benefits regarding care management, decision making and ability to implement risk‐reduction lifestyle changes [17].

In terms of clinical practice, in the USA, both the Alzheimer's Association and American Academy of Neurology now recommend annual cognitive screening for individuals aged 65 years or older [18, 19], despite a recent study demonstrating that dementia screening does not improve quality of life, healthcare utilisation or advanced care planning [15]. Given the disconnect between the evidence and the policy, considering the perspectives of primary and community care practitioners on the topic is important to understand services currently provided and the requirements for the future, should a robust base of empirical evidence emerge for dementia screening.

The term dementia screening can be used inconsistently, often referring indiscriminately to detection in asymptomatic individuals and early diagnosis in symptomatic individuals [4]. It is important to differentiate between three distinct areas: population screening, dementia case‐finding and dementia risk screening (see Table 1 for definitions), as different tools and approaches (e.g. blood markers, digital endpoints) may fall under different remits. Our aim with this review was to update Martin, et al. [14]'s systematic review by re‐examining the attitudes and preferences of healthcare professionals regarding primary care and community‐based dementia screening with a focus on the three distinct approaches: population screening; dementia case‐finding and dementia risk screening.

**TABLE 1:** Table of definitions regarding different types of dementia screening.

Type of screening	Definition
Population screening	A secondary prevention method which involves identifying an illness, such as dementia, amongst a population of apparently asymptomatic individuals. This excludes identifying dementia in people with pre‐dementia syndromes, such as mild cognitive impairment or subjective cognitive decline, or with chronic conditions associated with incidence of cognitive impairment, such as Parkinson's disease.
Case‐finding	A secondary prevention method whereby individuals are assessed for dementia at a point where they have a clear and higher probability of the condition (e.g. older age, risk factors, family history), or those who in the clinical opinion of the GP would benefit from the assessment.
Dementia risk screening	A primary prevention method which involves individuals being screened for risk factors of dementia (e.g. poor sleep, heart disease) and provided a risk score or equivalent regarding their dementia risk.

*Note:* Definitions taken from Ranson, et al. [4].

## 2 Methods

This systematic review was pre‐registered on PROSPERO (CRD42024531455) and followed the Preferred Reporting Items for Systematic reviews and Meta‐Analyses (PRISMA) guidelines (see Supporting Information S1) [20].

### 2.1 Search Strategy

Six electronic databases were used for this search, including Ovid Medline, Ovid Embase, Ovid PsychInfo, CINAHL, CRD and Cochrane Library. An example of the search strategy is provided in Supporting Information S2. All research between 01.01.2012 and 24.02.2024 was therefore considered for this review.

### 2.2 Eligibility Criteria

Table 2 details the review eligibility criteria. We included articles focussed on population screening, case finding and dementia risk screening.

**TABLE 2:** Eligibility criteria for article selection in this review.

Factors	Inclusion criteria	Exclusion criteria
Language	English‐written articles	n.a
Time frame	Published in August 2012	Published prior to August 2012
Location/setting	Primary and community care settings only (as defined by the study itself to allow for cultural differences)	Secondary and tertiary healthcare services
Topic	Dementia screening, characterized as population screening, case finding and dementia risk screening	– Attitudes towards screening for mild cognitive impairment (who do not meet the criteria of dementia) as this is a clinically contentious label [21]
		– Attitudes towards early or timely diagnosis. These concepts relate to the identification of prodromal or clinical dementia when the individual is starting to present or is fully presenting symptoms [22]
Intervention/Exposure	Tests that can currently be easily administrated and using variables that can be easily ascertained in primary or community care settings to screen for dementia e.g. electronic, pen and paper, biomarkers.	– Any tests that cannot be easily carried out in primary care e.g. genetic tests, lumbar puncture, amyloid PET imaging
		– Screening tools to detect persons with mild cognitive impairment
Comparator/Control	Any comparator or no comparator	n.a
Population	Health and social care professionals' perceptions, views and experiences will also be included.	– Studies using samples from the general public, people living with dementia and carers (informal and formal) who are not health and social care professionals.
Study type	All study types (qualitative, quantitative and mixed methods)	Case studies
Publication type	Peer reviewed publications written in English	– Unpublished sources
		– Opinion based papers
		– Conference abstracts
		– Reviews

### 2.3 Selection Process

**FIGURE 1:**
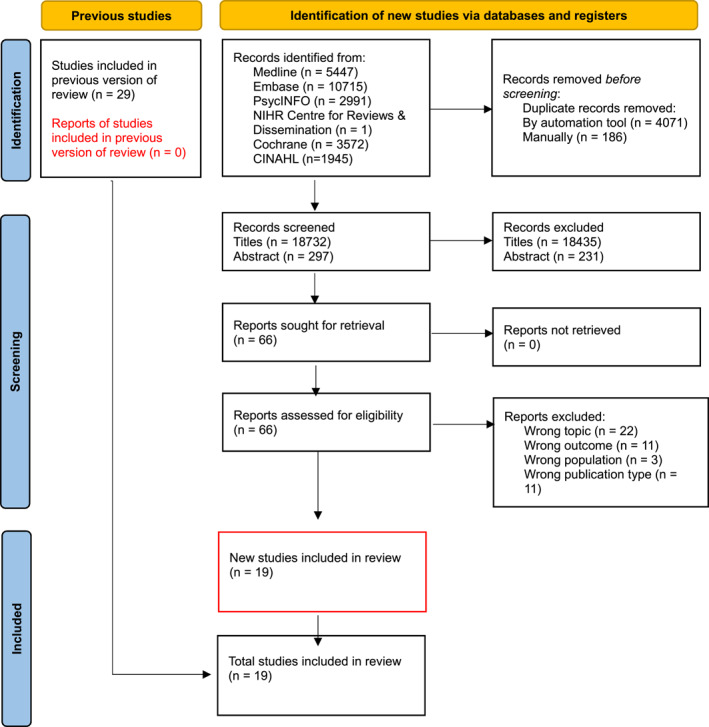
PRISMA diagram demonstrating the flow of study selection for the systematic review.

All references were exported to Endnote and duplicates were removed using automation tools, followed by a manual hand‐search. All de‐duplicated references were exported to Rayyan software for screening. Two reviewers (RMA and AD) independently screened titles, abstracts and full texts were independently screened by two reviewers. Disagreements were resolved through a two‐stage process: (1). Where possible, the reviewers discussed conflicting articles and came to an agreement, and (2). When an agreement could not be reached, a third independent author settled the disagreement (LR). Reasons for exclusion were recorded during the full text screening stage. Details of the selection process can be found in Figure 1. Following data extraction, an additional hand‐search was conducted to yield any remaining eligible articles.

### 2.4 Data Extraction

Data extraction forms were developed on Excel (Microsoft Corp) by RMA, in consultation with AD. Key measures of interest were extracted from all eligible articles, including: study aim, setting (e.g. primary/community care), geographical location, sample demographics, type of health and social care professional (e.g. GP), dementia screening method, and key results relating to health and social care professionals' perspectives regarding dementia screening. Although the perspectives of healthcare professionals did not need to relate to a specific screening programme, authors RMA and AD reviewed all descriptions of dementia screening and classified them against the three areas of interest: population screening, case‐finding and dementia risk screening.

### 2.5 Quality Assessment

The ‘Mixed Methods Appraisal Tool’ (MMAT) was used to appraise the quality of included articles, which is suitable for their mixed methods nature [23]. Articles were independently assessed by two authors (RMA, AD) and discrepancies resolved by discussion. The MMAT discourages calculating an overall score for quality, and recommends presenting the details of ratings for readers' discretion; we have included this in Supporting Information S3.

### 2.6 Data Synthesis

A narrative data synthesis was conducted whereby quantitative and qualitative data were integrated according to a convergent integrated approach [24]. This involves ‘qualitising’ quantitative data, whereby extracted data is converted into textual descriptions to allow integration with qualitative data. All data was considered and discussed by team members (RMA, AD, LR) and multiple codes were assigned. Data was reviewed in an iterative process until core categories were identified based on meaning; these formed overarching themes and subthemes within and are presented as such.

## 3 Results

As shown in Figure 1, 18,732 articles were identified from the electronic search following removal of duplicates. Sixty‐six were included for full text screening following title and abstract review. Eleven conflicts emerged during title screening, nine of which were rectified via discussion and two adjudicated by a third reviewer. Nineteen articles met the inclusion criteria and are reported in this review. Included articles were published between 2013 and 2024. Studies were largely from high income countries, set in the following locations: USA (*n* = 6), United Kingdom (*n* = 4), Australia (*n* = 2), France (*n* = 2), Belgium (*n* = 2), Canada, Germany, Italy, Spain, Hong Kong, Japan, Hungary, Poland, China, and Taiwan (*n* = 1 for all). Eighteen studies took place in primary healthcare settings, as defined by the study to account for cultural differences between countries, while one was based in a community setting. Quality appraisal of all studies can be found in Supporting Information S3.

Eight articles presented findings from qualitative research, nine from quantitative and two from mixed methods. Seventeen articles included perspectives or attitudes of primary care physicians or general practitioners. To ensure consistency in terminology, we refer to both as primary care practitioners (PCPs) throughout the results; study‐specific terminology can be found in Table 3. From the remaining articles, one reported on the perspective of community nurses and the other community physical therapists. Sample sizes ranged from 10 to 1365. Further demographic information for each study can be found in Table 3.

**TABLE 3:** Demographic information and key results of all eligible studies.

Authors	Type of screening	Setting	Demographics	Methods	Key results
Abe et al. (2021)	Population screening	Primary care services, Japan and USA	**Japan** Rural–12 primary care physicians 75% male Years in practice: 12.7 (SD: 5.9; range: 6–24) Urban–12 primary care physicians 67% male Years in practice: 17.4 (SD: 8.1; range: 9–38) **USA** Rural–12 primary care physicians 42% male Years in practice: 7.9 (SD: 6.6; range: 2–23) Urban–12 primary care physicians 42% male Years in practice: 19.3 (SD: 9.0; range: 6–34)	**Qualitative** Semi‐structured interviews	**Perspectives** USA physicians: Dedicated screening services allow prompts for further screening. Early detection can support advance care directives and care planning. **Barriers to screening** Not supported **Facilitators/requirements for screening** Not supported
Bandini et al. (2022)	Population screening	Primary care services, USA	11 primary care providers 82% primary care physicians 18% primary care nurse practitioners 64.6% male 63.6% non‐hispanic white 27.3% Asian 9.1% other Years since training: 12.1 years (SD: 10.7)	**Qualitative** Semi‐structured interviews	**Perspectives** Not reported **Barriers to screening** Lack of standardised screening tools or processes. Lack of available treatment. Time concerns Competing interests. Language/educational barriers. Concern over overwhelming secondary services. **Facilitators/requirements for screening** Self‐administered screener.
Chithiramohan et al. (2019)	Population screening	Primary care services, UK	13 general practitioners 62% male GP experience range: Newly qualified to 26 years Participant ethnicity: White European, Arab, Chinese, Black African, British Indian, British Sri Lankan, Pakistani and mixed.	**Qualitative** Semi‐structured interviews	**Perspectives** Opportunistic screening considered impractical due to lack of time. Screening tools are designed to highlight red flag symptoms, and diagnosis is for secondary care. **Barriers to screening** Lack of time Competing interests Language/educational issues. Inefficient referral management and co‐ordinate with secondary services. Lack of treatment. Lack of incentive. Lack of physician interest. Physician hesitancy due to worry about offending the patient. Limited frequency of patient consultations. Patient lack of awareness of dementia or reluctance in help‐seeking. **Facilitators/requirements for screening** Training in dementia screening and symptom recognition. Physician–patient relationship with strong rapport
Chmiela et al. (2023)	Population screening	Primary care services, Poland	175 primary healthcare doctors 46 male Age (years): 46 (SD: 14) Years of work in primary healthcare: 16 (SD: 13) 54.86% family medicine specialists 32% Internal medicine specialists 18.29% different specialisation 18.29% No specialist title	**Quantitative** Questionnaire	**Perspectives** 91.4% see advisability of dementia screening in > 65 years. 30.29% believe dementia screening should be conducted by GPs. Physicians without specialisation more likely to believe screening should take place in secondary services. 41.4% willing to participate in a programme to support dementia screening. **Barriers to screening** Lack of time–83% suggested screening needs to take < 10 min. **Facilitators/requirements for screening** Not reported
Crombie et al. (2024)	Population screening	Primary care services, Australia	**Stage 1**: 16 general practitioners 7 male Clinical practice experience: 1–30 years **Stage 2**: Pre‐training–14 general practitioners 8 male Post‐training–10 general practitioners 5 male Years working in general practice: 18 months to over 30 years	**Mixed methods** Semi‐structured interviews Pre and post training surveys	**Perspectives** An educational training programme led to increased belief that early detection benefits patients, increased the number of physicians who actively search for dementia in patients overy 65 years old or who use cognitive tests when suspicious of cognitive problems, and a decrease in belief that early detection has no therapeutic consequences. **Barriers to screening** Lack of time Patient reluctance **Facilitators/requirements for screening** Dedicated service–most found the 75+ health assessment a non‐threatening way to prompt screening for memory issues.
Gaboreau et al. (2014)	Population screening	Primary care services, France	493 general practitioners 66% male Age: 49.96 (SD: 8.47) Length of medical practice: 20.45 (SD: 9.11)	**Quantitative** Questionnaire	**Perspectives** 73.2% agreed annual screening was useful. **Barriers to screening** Younger age in physicians Region‐specific Negative perception of utility of dementia screening. Social difficulties in medical care of 75 year olds. Lack of time Lack of knowledge Unsuitable working conditions Lack of financial compensation Insufficient technical resources Insufficient motivation Patient non‐compliance Forgetting to screen. Lack of interest in dementia screening **Facilitators/requirements for screening** Greater interest in geriatrics and dementia Increasing clinical visit frequency Higher percentage of patients over 75 years
Judge et al. (2019)	Population screening	Primary and secondary care services, Canada, France, Germany, Italy, Spain, UK, USA.	1365 primary care and specialist physicians 37% primary care physicians	**Quantitative** Survey	**Perspectives** Physicians in the USA were more likely to perform routine cognitive testing as part of an annual wellness visit, compared to physicians in other countries (25% vs. 8%) **Barriers to screening** Not reported **Facilitators/requirements for screening** Not reported
Gong et al. (2023)	Dementia case‐finding	Primary care services, China	52 general practitioners 50% male Mean age: 31.3 (range: 24–46) Mean years in practice: 6.6 (range: 1–28) Focus group: 30 GPs (10 in each group) Interviews: 22 GPs	**Qualitative** Interviews and focus groups	**Perspectives** Not reported **Barriers to screening** Poor knowledge and skills of screening and relevant guidelines for advice and referral Inadequate screening skills Unavailable resources Lack of continuity in screening due to migratory population Unclear pathways for referrals. Lack of time. Competing demands. Lack of dementia specialists. Low motivation Belief dementia screening is for secondary services Lack of confidence. Worries regarding dementia stigma Insufficient financial incentives. **Facilitators/requirements for screening** Dedicated service/time for screening Different terminology for dementia screening i.e. health screening.
Huang et al. (2013)	Dementia case‐finding	Community services, Taiwan	195 community health nurses 98% female Mean age: 35.4 (SD: 6.5) Years working as a nurse: 11.5 (SD: 6.6)	**Quantitative** Questionnaire	**Perspectives** Not reported **Barriers to screening** Not reported **Facilitators/requirements for screening** Higher education and experience Greater confidence regarding dementia care Job role–care managers and home health care nurses more likely to engage than district nurses.
Jan et al. (2021)	Dementia case‐finding	Primary care services, Belgium	331 general practitioners 45% male Age: 21–29: 12.08% 30–39: 32.02% 40–49: 14.8% 50–59: 21.75% 60–64: 12.69% > 65: 6.65%	**Quantitative** Survey	**Perspectives** Not reported **Barriers to screening** Lack of time Lack of knowledge Physician age–older physicians felt diagnostic expertise inadequate Difficulties referring patients to secondary services **Facilitators/requirements for screening** Financial renumeration Help from dementia expert physicians
Leung et al. (2020)	Dementia case‐finding	Primary care services, Hong Kong	**Focus groups** 31 primary care physicians 41.9% male 55% more than 11 year of work experience after graduating. **Questionnaire** 437 primary care physicians 60.8% male 22.4% more than 30 years of work experience 96.7% primary care	**Mixed methods** Focus group Questionnaire	**Perspectives** Felt they lacked competence to detect dementia due to lack of experience and felt a specialist should be involved. Agree they play an essential role in screening. Belief that early detection leads to communication of information and delivery of treatment. Concerned that diagnosis may be false positive or the label may lead to added stress. Need a follow‐up plan to avoid stigma and anxiety. Most felt primary care physicians had an important role to play. **Barriers to screening** Not reported **Facilitators/requirements for screening** Geriatric or psychogeriatric training
Miles et al. (2019)	Dementia case‐finding	Community services, USA	233 home care physical therapists 53 male Median age: 49 (range: 25–77) Median years working in PT: 23 (range: 1–55) 13%: 10 years or less PT experience 31.8%: More than 31 year of PT experience	**Quantitative** Questionnaire	**Perspectives** 90% felt physical therapists were qualified to administer cognitive screens and most felt comfortable to do so. **Barriers to screening** Not reported **Facilitators/requirements for screening** Not reported
Schoenmakers & Robben. (2021)	Dementia case‐finding	Primary care services, Belgium	122 general practitioners 64.8% female	**Quantitative** Survey (*Only quantitative‐survey relevant to this review*)	**Perspectives** Not reported **Barriers to screening** Language barriers Patients not presenting with cognitive complaints Difficulty discussing subject openly Need for hetero‐anamnesis Illiteracy Family unwilling to accept investigation. Inappropriate screening tools. **Facilitators/requirements for screening** Not reported
Suchsland et al. (2023)	Dementia case‐finding	Primary care services, USA	10 primary care physicians 9 female 9 physicians, 1 nurse practitioner Clinical experience range: 4.5–28 years	**Qualitative** Semi‐structured interviews	**Perspectives** No clear method to determine if participants would benefit from a cognitive evaluation. **Barriers to screening** Lack of standardised screening processes. Lack of time Patient reluctance/anxiety Provider hesitation Concern over availability/accessibility of referring to secondary services. **Facilitators/requirements for screening** Dedicated screening services Easy to do. Integrated into time allotted for the visit.
Balogh et al. (2020)	Dementia case‐finding	Primary care services, Hungary	402 general practitioners Gender (387 GPs) 46.3% male Age (393 GPs) 25–35: 5.9% 36–45: 12.5% 46–55: 24.9% 56–65: 40.2% > 65: 16.5% Place of practice (372 GPs) 66.1% urban *(Completion rate varied for each question)*	**Quantitative** Questionnaire	**Perspectives** 90% felt early therapy could slow symptom progression. 97% felt early detection enhances both the patient and relatives' wellbeing. 68% agree screening in primary care could lead to more effective outcomes in therapy 79% would implement screening tests if conditions were suitable **Barriers to screening** Not reported **Facilitators/requirements for screening** More clinical time Up to date tests Help from assistants and more staff More exam rooms.
Lathren et al. (2013)	Dementia case‐finding	Primary care services, USA	29 primary care physicians 59% male Mean age: 52 (SD: 8.3) Speciality 65% family practice 35% Internal medicine 21% geriatrics subspeciality Mean years in practice: 20.8 (SD: 8.6)	**Quantitative** Pre training interview and follow up questionnaires	**Perspectives** Not reported **Barriers to screening** Not reported **Facilitators/requirements for screening** Knowledge and skills–following an educational training programme: Increase in confidence for screening participants. Increase in use of dementia clinical screening and assessment. Influenced who they screened–screening more based on participants' age. Change in use of screening procedures.
Godbee et al. (2020)	Dementia risk screening	Primary care services, Australia	13 general practitioners 7 female 4 general practice nurses 4 female	**Qualitative** Semi‐structured interviews	**Perspectives** Physicians feel there is sufficient evidence to give general advice on dementia risk reduction, with good knowledge on risk factors and preventative messaging. Some believed it is adequate to manage dementia risk by managing cardiovascular and diabetes risk as mentioning dementia may distress patients. **Barriers to screening** Lack of time Lack of appropriate tools Insufficient technical resources Insufficient information to provide patients. Competing demands Lack of financial renumeration Lack of confidence Poor communication between clinical staff. Lack of methods to measure effectiveness of promoting risk reduction. Lack of familiarity with patients. Lack of standardised opportunity to discuss risk **Facilitators/requirements for screening** Availability of secondary services Fits well within existing systems Access of patients to preferred doctor Frequent follow‐up appointments
Jones et al. (2024)	Dementia risk screening	Primary care services, UK	11 general practitioners 9 female Time qualified as GP < 5: 5 years 6–20: 1 year > 20: 5 years	**Qualitative** Semi‐structured interviews	**Perspectives** Physicians acknowledge need to play an active role in dementia prevention. **Barriers to screening** Competing demands. Lack of time. Lack of knowledge and skills. Fear and stigma of dementia/physician hesitation **Facilitators/requirements for screening** A whole team approach. Dedicated screening services. Public health messaging. Information provision Alternative terminology e.g. brain health
Wilson et al. (2023)	Dementia risk screening	Primary and secondary care services, UK	11 general practitioners 6 male Years in the NHS range: 10–35 (*Only primary healthcare professionals included)*	**Qualitative** Semi‐structured interviews	**Perspectives** Not reported **Barriers to screening** Concerns on overburdening healthcare services **Facilitators/requirements for screening** Appropriate screening tools Dedicated screening services Additional space and funds Additional secondary services Knowledge on evidence behind the screening tool and risk score. Mindfulness of inequities.

**FIGURE 2:**
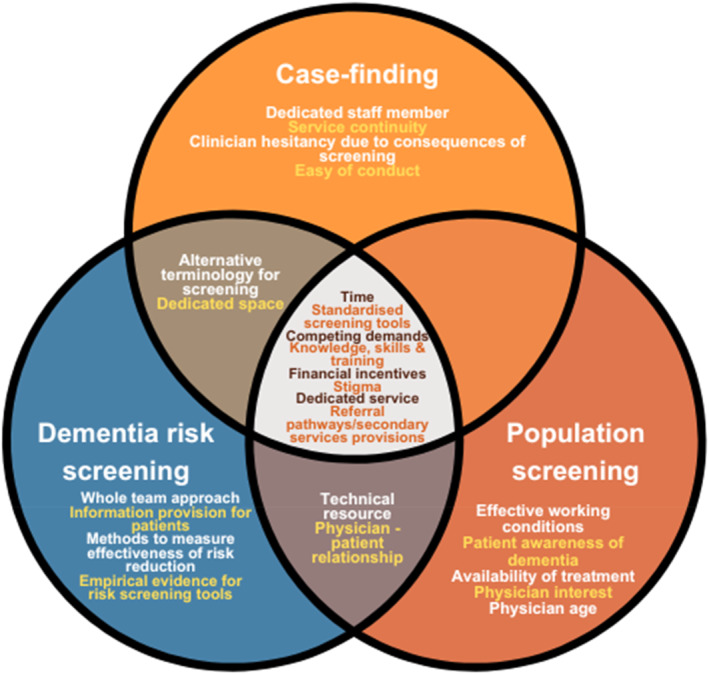
Venn diagram highlighting commonalities and unique influences (i.e. barriers and/or facilitators/requirements) on healthcare professionals' perceptions of different types of dementia screening.

Regarding dementia screening, nine studies were concerned with dementia case‐finding, seven with population screening and three with dementia risk screening. Current practices, perceptions, barriers and facilitators/requirements for each kind of screening are presented. Key results of each paper can be found in Table 3 and a synthesis of the common and unique influences on each kind of screening is illustrated in Figure 2.

### 3.1 Population Screening

PCPs in three studies in the USA, Australia, and France indicated engaging with population screening in current practice, although primarily through a dedicated service (e.g. Annual Medicare Wellness checks, 75+ health assessment) [25, 26, 27]. One large international study, (USA, Canada, France, Italy, Germany and the UK) indicated that PCPs in the USA were more likely to perform routine cognitive screening as part of dedicated services than the other countries [28]. These dedicated services offered mechanisms to address cognitive concerns with patients and initiate further cognitive screening without introducing further stigma [25, 29].

Regarding perspectives on population screening, the majority of PCPs in two studies agreed it was useful to screen older patients for cognitive issues [27, 30] but less than half of the PCPs in one study indicated that they would be willing to participate in the implementation of a screening programme [30]. PCPs in two studies agreed that screening could be beneficial for support advance directives, care planning [28] and quality of life [31], while those in two studies noted issues around screening pertaining to lack of treatment, its impractical nature and concerns about overburdening secondary services [26, 31]. One study also suggested that PCPs mostly felt population screening should be conducted by secondary services [30]. One study in Australia, which conducted a single day educational dementia training programme for rural PCPs (covering dementia recognition, risk factors and management, behavioural symptoms, carer stress, support services, legal issues, end of life issues), led to increases in beliefs around benefits of proactive detection (71% vs. 100% pre‐post agreement), and changes in current practice–for example actively searching for dementia in patients over 65 years (36% vs. 100% pre‐post agreement) [29].

Barriers to population screening include resource requirement, patient‐related factors, unclear post‐diagnostic pathways, communication issues and physician‐led factors. Four studies reported lack of time to conduct screening [26, 27, 30, 31], while other resource issues included lack of standardised tools or processes [26] (particularly regarding those with low education or minority cultural/ethnic groups [26, 31]), limited financial incentives as a barrier to screening [27, 31], unsuitable working conditions and insufficient technical resources [27]. Two studies further considered competing demands as a barrier, particularly regarding the ‘hierarchy’ of disease (e.g. prioritising diabetes screening over dementia) [26, 31]. Patient‐related factors were mentioned in two studies, including stigma, lack of dementia awareness [31] and reluctance of the patient or family to address concerns [30]. Regarding post‐screening, lack of treatment for dementia was cited as a barrier. Regarding communication, inefficient referral management and communication with secondary services was a cause of concern [31]. For physician‐led factors, two reported lack of interest as a barrier [27, 31], two suggested lack of knowledge regarding screening or screening tools [27, 30].

Facilitators, incentives and requirements were common across studies, including need for standardised tools, familiarity with the patient [31] and time, whereby the majority of PCPs in one study agreed a screening test should take < 5 min to conduct [30].

### 3.2 Dementia Case‐Finding

From three studies based in Belgium, Taiwan and Hungary, most PCPs (specifically GPs or community health nurses) were currently practising or had practised case‐finding in their services, although standard cognitive tests or symptom lists were not commonly used [32, 33, 34]. Additionally, most PCPs in one study included questions about risk factors in their history taking [32].

Regarding perceptions of practice, two studies reported that PCPs regarded case‐finding as beneficial to patients; it facilitated earlier information provision, intervention and treatment, and time to support decision‐making [33, 35]. Three studies reported negative perceptions, namely regarding perceived low efficacy of case‐finding [36], negative consequences for patients including legal consequences and psychological distress [35], and lack of cost‐efficacy [35] and clarity regarding benefits [37]. Additionally, two studies reported worries about misdiagnosis or false positive results [36]. Two studies reported practitioners as considering dementia case‐finding within their role as either PCPs (*n* = 1) or physical therapists (*n* = 1) [35, 38], while two suggested it as a role for specialist and secondary services [35, 36]. Additionally, two studies reported the need for a dedicated member of staff with dementia expertise to conduct or facilitate case‐finding [32, 36].

Common barriers were discussed across studies: these included knowledge, skills and confidence, resource requirements and patient‐related barriers. Four studies mentioned a lack of knowledge, skills and confidence regarding dementia and case‐finding as a barrier [32, 35, 36, 37], noting particularly the lack of guidelines around both the screening and referral processes [32, 36], uncertainty regarding post‐screening actions and insufficient training in the area [35, 37]. Four studies mentioned lack of resource as a barrier to case‐finding [32, 35, 36, 37]; all of them mentioned lack of time to conduct case‐finding and associated activities [32, 35, 36, 37]. Pressure from competing demands was also mentioned by two studies, referring to screening and management of other chronic conditions such as hypertension and diabetes [36, 37]. Lack of standardised or appropriate tools were also mentioned by three studies, referring to cognitive screening tests [35, 37, 39]. There were additional concerns regarding the lack of secondary resources, such as accessibility to social care or community resources [37]. Patient‐related barriers were also mentioned in four studies, particularly regarding lack of help‐seeking behaviour from patients due to the stigma surrounding dementia [36, 39], concealment of cognitive symptoms [36, 39], refusal to engage in screening activity [39], and migration of patients leading to lack of service continuity [36]. Additionally, PCPs discussed worries about broaching the topic of case‐finding due to negative perceptions and emotions regarding dementia from patients [35, 36, 37], preferring to use alternative terminology for the subject such as ‘health screening’ [36].

Common facilitators, incentives or requirements for engaging in case‐finding included knowledge and skills, additional resource and provision of a dedicated service and post‐screening pathway, and financial renumeration. Two studies reported that PCPs with more education and experience appeared more confident and likely to engage in case‐finding behaviours [34, 38]. One study demonstrated that an educational intervention, conducted in collaboration with local support service providers and focussing on evidence‐based information regarding screening, diagnosis and assessment, treatment and management, caregiving an community resources, led to higher confidence in conducting case‐finding, particularly regarding who to screen [40]. Two studies indicated that case‐finding needs to be easy to do and require little time cost [33, 37], with up‐to‐date appropriate tests and greater staff and space resource [33]. Two studies discussed the requirement of a special time or dedicated service needed to conduct case‐finding in a targeted population [36, 37], with the Medicare Annual Wellness Visit used as important example [37]. Additionally, one study stressed the need of a follow‐up plan or pathway to ensure positive outcomes from case‐finding [35], while another regarded financial incentives as key motivators or requirements to engage in case‐finding practice [36].

### 3.3 Dementia Risk Screening

Two studies in Australia and the UK reported mixed results regarding engagement of PCPs in dementia risk screening within current practice; although some PCPs were beginning to promote dementia risk reduction, this was not widely or systematically applied [41, 42]. The majority of PCPs never explicitly discussed dementia risk with patients, despite acknowledging the importance of their role in the prevention of dementia and other chronic conditions [41, 42], except where patients' raised specific concerns regarding dementia [41].

Regarding perceptions of dementia risk screening, PCPs agreed that it was important for providing information regarding dementia prevention and supporting lifestyle changes, which could also aid prevent other conditions [41, 42]. One study reported that PCPs felt there was sufficient empirical evidence surrounding dementia risk reduction to engage in this screening behaviour [42]. Another study reported concerns from PCPs that dementia risk screening would overburden the healthcare services due to increasing demands on primary and secondary care from ‘at‐risk’ individuals who may still be asymptomatic [43]. Regarding service delivery, two studies felt that dementia risk screening should be part of a dedicated risk reduction service such as annual health checks, long‐term condition or medication reviews [41, 43], while one study reported that PCPs felt dementia risk screening would fit within the existing primary care service but that chronic disease management and health assessments also offered opportunities for the service [42]. PCPs in one study suggested this type of screening required a whole team approach for greatest impact, including physician associates, nurse practitioners, pharmacists, paramedics and other allied health professionals [41]. This fits with discourse in two studies regarding the importance of good communication between clinical teams to deliver dementia risk screening services [42] and the need for whole primary care teams to be involved in future research, education and implementation [41].

Common barriers for dementia risk screening related to professional knowledge and skills, resource requirements and patient‐related barriers. Two studies reported that PCPs felt they lacked the knowledge and skills to conduct dementia risk screening, particularly regarding information to promote dementia risk reduction in terms that patients would understand without being overwhelmed [41, 42]. Resource issues included lack of time to conduct screening and follow‐up consultation [41, 42], appropriate screening tools [42, 43], appropriate computing systems and prompts [42], financial incentives for PCPs [42], appropriate information to provide patients following screening [42] and methods to measure how well PCPs are promoting dementia risk reduction and monitoring the implementation of lifestyle changes in patients [42]. Additionally, two studies highlighted lack of resource due to competing demands as a barrier, with dementia risk screening a lower priority for preventative care focussed on other chronic conditions [41, 42]. Patient related barriers included practitioners' reluctance to mention dementia due to related stigma and concern that it would cause patient anxiety [41, 42]. PCPs preferred terms such as ‘brain health’ to reduce negative connotations [41].

Facilitators, incentives and requirements were mentioned in each of the three studies, namely communication, post‐screening pathways, resource requirements, and empirical evidence. Three studies considered communication, PCPs in one study felt that communicating risk of dementia would be no more difficult than that of other chronic conditions [42], practitioners in another study highlighted the need to understand and communicate how a risk score was generated to inform patients regarding lifestyle changes [43] and the third advised that health promotion materials such as leaflets would alleviate the time cost of screening [41]. Two studies outlined post‐screening requirements whereby PCPs in one study highlighted that the availability of community supports for dementia risk reduction would incentivise screening [42] while another highlighted the need for post‐screening pathways relating to psychological and practical support for those at higher‐risk [43]. Regarding resource, one study highlighted patients' ability to access their preferred familiar doctor would be important for adoption of risk screening [42] while PCPs in another set requirements for additional space to deliver a new screening service and funding for new treatments to reduce dementia risk [43]. PCPs in one study required empirical evidence regarding the accuracy and validity of the dementia risk screening tools to consider adoption [43].

## 4 Discussion

To reflect the changing ethos in dementia research, policy and practice, this systematic review updated earlier work to re‐examine the perspectives and attitudes of healthcare professionals from primary and community care backgrounds regarding different aspects of dementia screening. Capturing these perspectives, particularly regarding acceptability, is a fundamental criterion to the implementation of any screening programme in accordance with international criteria [44, 45, 46, 47, 48]. The majority of included articles were from high‐income countries. Key findings reveal mixed views regarding the benefits, and willingness to conduct population screening or case‐finding for dementia, although PCPs appear interested in engaging with dementia risk screening and risk reduction efforts according to the limited studies on this approach. Common influences on professionals' perceptions included the knowledge, skills and training of the healthcare professional, provision of appropriate resources and clinical time, provision of a dedicated service to conduct screening, patient‐related factors regarding dementia stigma, and ability to refer patients to secondary services following screening.

It has been over a decade since Martin, et al. [14]'s systematic review of this topic, and significant advancements have been made regarding our understanding and policies regarding dementia risk detection and reduction in that time [3, 4, 7, 8, 10, 12, 13, 49]. We tailored our systematic review to reflect these changes, particularly by considering the different types of dementia screening that have been introduced into the literature and clinical practice in the interim. However, our key findings regarding perceived barriers of dementia screening from the healthcare professionals' perspectives bear striking similarities to the original review [14]. Screening is known to also cause harm as well as benefit for a variety of reasons [50]. Many of these were identified by health care professionals (e.g. unintended consequences). Healthcare professionals are still unclear regarding benefits of all three approaches to dementia screening, do not feel there are adequate clinical resources, funding, screening tools or allowances on clinical time to conduct screening, have competing demands due to the hierarchy of managing more treatable medical problems, are concerned regarding the impact of screening on patients given the stigma attached to dementia, and unclear regarding access or availability of post‐screening pathways for patients. This suggests an important disconnect between current policies and recommendations [1, 3] and the capability and capacity of primary healthcare services to meet these policies and recommendations, largely at an organisation and contextual level.

Interestingly, the views of healthcare professionals depicted in this review contrast significantly from those captured in a recent systematic review regarding the perspectives of lay people from predominately USA‐based ethnic minority backgrounds [17]. The lay perspective generally viewed dementia screening as beneficial to decision‐making, implementation of lifestyle changes and access to treatment and interventions, although the dementia screening approach was not specified [17]. This discrepancy may reflect a more pragmatic understanding of dementia screening by professionals, whereby the patient and cost benefit have yet to be shown [46, 47, 48, 50]. For example, the only randomised control trial to investigate utility of dementia screening showed no improvements in patients' health‐related quality of life following screening, along with low uptake of further cognitive assessment for those deemed ‘at risk’ and overall low diagnostic rates [15]. Notably, the trial indicated no evidence for either benefit or harm from dementia screening. There is also still a lack of evidence for effective markers of prodromal dementia and treatments to significantly slow or halt disease progression [50]. Healthcare professionals included in the eligible studies often highlighted lack of knowledge about dementia as a condition and the benefits and consequences of any approach of dementia screening as an influence on their perceptions of dementia screening, but it may be that the evidence does not currently exist. A future research trial could consider offering multiple different modules offering varying levels of evidence regarding dementia screening, and considering the cost‐benefit of different service provision models (e.g. dedicated service vs. usual healthcare advice).

Results suggest that primary healthcare professionals hold more positive perceptions of two areas of dementia screening: use or provision of a dedicated dementia screening service and screening to support dementia risk reduction. Dedicated dementia screening services were discussed in relation to each of the three approaches, and likely reflects the professionals' need to have dedicated time and resource for the task. Several papers discussed current practices of screening for dementia during dedicated health screening services (e.g. Medicare's Annual Wellness Visit), suggesting this provides sufficient time to conduct screening, and allows a discussion about cognitive issues without introducing stigma or negative emotions from the patient [25, 26, 27, 28, 36, 37, 41, 43]. Additionally, these screening services often consider screening for other chronic conditions and facilitated the use of less stigmatising terminology for example ‘brain health screening’ as opposed to ‘dementia screening’ [41]. These findings were not identified in Martin, et al. [14] and likely reflect initiatives that have occurred since then; for example, recommendations to cognitively screen all 65+ years olds in the USA by the Alzheimer's Association in 2013 and American Academy of Neurology in 2019 [18, 19]. Future initiatives, such as dementia risk prediction indices [51], should be integrated within existing health screening services, for example part of annual health check, to improve acceptance and uptake from healthcare professionals, if their evidence base supports wider implementation [50, 52].

Importantly, healthcare professionals appear to support screening for dementia risk (i.e. identifying those at higher dementia risk due to a weighted risk score), largely to support patients with lifestyle changes which may also have more immediate benefits on other chronic conditions (i.e. diabetes, hypertension) [29, 42]. This suggests more broadly, from a limited sample of three studies, that healthcare professionals may find engaging with primary prevention approaches to dementia as acceptable. There are two approaches to primary prevention: identifying individuals at high‐risk of developing a condition and using individual‐level interventions to reduce risk, or lowering risk at a population level through wider policy changes [53]. Although the high‐risk individual level approach has been favoured in dementia research, a recent study using a longitudinal, broadly representative, sample with up to 29 years of linked health data reported that 70% of all dementia cases arose from those with ‘normal risk’, suggesting that most dementia cases would be missed using this approach [54]. Additionally, 80% of people in the ‘high‐risk’ group did not go on to develop dementia within the time frame of the study. Based on this evidence, Walsh, et al. [54] suggests that screening for dementia risk may not lend itself to meaningfully reduce dementia incidence at a population level. However, healthcare professionals could provide population‐level information on risk reduction techniques to all patients without the need for disease‐specific screening, and evidence from this review suggests interest in engaging with risk reduction conversations [29, 42].

Strengths of this systematic review include a pre‐registered protocol and a comprehensive search strategy and selection process for inclusion of eligible articles, aligned with PRISMA guidelines [20]. Different approaches to ‘dementia screening’ were considered discretely, allowing nuances to examined within the results and interpretation. Limitations of this review include a lack of articles from low‐middle income countries on this topic. Given that 61% of people with dementia live in low‐middle income countries, it is crucial to include the perspectives of healthcare professionals regarding screening in those regions [55]. Our search and eligibility criteria may have had limited ability to identify relevant papers (e.g. limited to English‐written articles due to resource); however, it is also possible that research on this topic has not matured at the same rate as in high‐income countries, for example due to the lack of culturally‐appropriate screening tools available to these regions or competing research priorities [56, 57]. Finally, with the exception of two studies which detailed their educational programmes featuring dementia screening, it is unclear what information participants in the eligible studies were provided with regarding the topic prior to providing their perspectives.

## 5 Conclusion

To conclude, our systematic review found mixed perspectives from predominately PCPs regarding the benefits of and willingness to conduct population screening and case‐finding for dementia, although attitudes towards dementia risk screening were more positive. Influences (i.e. barriers/facilitators/requirements) on healthcare professionals' perspectives were mostly congruent across the types of dementia screening and reflected the findings of the previous Martin, et al. [14]'s review. Findings suggest that healthcare professionals' perspectives, capabilities and organisational structures are not aligned with dementia policies around dementia screening, particularly the emphasis on case‐finding and dementia risk screening, and risk reduction. Recommendations from this review include embedding dementia screening into dedicated services such as health screening, should an empirical evidence base arise for their implementation, and to consider employing risk reduction approaches across the wider population in the absence of dementia screening.

## Conflicts of Interest

The authors declare no conflicts of interest.

## Supporting information

Supporting Information S1

Supporting Information S2

Supporting Information S3

## Data Availability

Data sharing is not applicable to this article as no new data were created or analysed in this study.
