# Supplementary material for: Attitudes and Preferences Towards Screening for Dementia From the Perspectives of Healthcare Professionals: An Updated Systematic Review
Source: Int J Geriatr Psychiatry. 2025 Feb 20;40(2):e70057. doi: 10.1002/gps.70057 (PMC11842158; doi:10.1002/gps.70057)
Supplement: Supplementary file 2 — Supporting Information S2 [file GPS-40-e70057-s001.docx]

**Previous studies**

**Identification of new studies via databases and registers**

Records removed *before screening*:

Duplicate records removed: By automation tool (n = 4071)

Manually (n = 186)

Studies included in previous version of review (n = 29)

Reports of studies included in previous version of review (n = 0)

Records identified from:

Medline (n = 5447)

Embase (n = 10715)

PsycINFO (n = 2991)

NIHR Centre for Reviews & Dissemination (n = 1)

Cochrane (n = 3572)

CINAHL (n=1945)

**Identification**

Total studies included in review

(n = 19)

Reports assessed for eligibility

(n = 66)

Reports sought for retrieval

(n = 66)

Records screened

Titles (n = 18732)

Abstract (n = 297)

Records excluded

Titles (n = 18435)

Abstract (n = 231)

Reports not retrieved

(n = 0)

**Screening**

Reports excluded:

Wrong topic (n = 22)

Wrong outcome (n = 11)

Wrong population (n = 3)

Wrong publication type (n = 11)

New studies included in review

(n = 19)

**Included**
