# Supplementary material for: Attitudes and Preferences Towards Screening for Dementia From the Perspectives of Healthcare Professionals: An Updated Systematic Review
Source: Int J Geriatr Psychiatry. 2025 Feb 20;40(2):e70057. doi: 10.1002/gps.70057 (PMC11842158; doi:10.1002/gps.70057)
Supplement: Supplementary file 3 — Supporting Information S3 [file GPS-40-e70057-s003.docx]

| **Mixed Methods Appraisal Tool (MMAT), version 2018** | | | | | | | | |
| --- | --- | --- | --- | --- | --- | --- | --- | --- |
| **Category of study design** | **Qualitative studies** | | | | | | | |
| **Author(s) of Corresponding Study(s)** | **Abe, et al. (2021)** | **Bandini, et al. (2022)** | **Chithiramohan, et al. (2019)** | **Godbee, et al. (2020)** | **Gong, et al. (2023)** | **Jones, et al. (2024)** | **Suchsland, et al. (2023)** | **Wilson, et al. (2023)** |
| Are there clear research questions? | Yes | Yes | Yes | Yes | Yes | Yes | Yes | Yes |
| Do the collected data allow to address the research questions? | Yes | Yes | Yes | Yes | Yes | Yes | Yes | Yes |
| Is the qualitative approach appropriate to answer the research question? | Yes | Yes | Yes | Yes | Yes | Yes | Yes | Yes |
| Are the qualitative data collection methods adequate to address the research question? | Yes | Yes | Yes | Yes | Yes | Yes | Yes | Yes |
| Are the findings adequately derived from the data? | Yes | Yes | Yes | Yes | Yes | Yes | Yes | Yes |
| Is the interpretation of results sufficiently substantiated by data? | Yes | Yes | Yes | Yes | Yes | Yes | Yes | Yes |
| Is there coherence between qualitative data sources, collection, analysis and interpretation? | Yes | Yes | Yes | Yes | Yes | Yes | No | Yes |

**Appendix 1 – Quality Appraisal**

| **Mixed Methods Appraisal Tool (MMAT), version 2018** | | | | | | | | | |
| --- | --- | --- | --- | --- | --- | --- | --- | --- | --- |
| **Category of study design** | **Quantitative descriptive studies** | | | | | | | | |
| **Author(s) of Corresponding Study(s)** | **Balogh, et al. (2020)** | **Chmiela, et al. (2023)** | **Gaboreau, et al. (2014)** | **Huang, et al. (2013)** | **Jan, et al. (2021)** | **Judge, et al. (2019)** | **Lathren, et al. (2013)** | **Miles, et al. (2019)** | **Schoenmakers, et al. (2021)** |
| Are there clear research questions? | Yes | Yes | Yes | Yes | Yes | Yes | Yes | Yes | Yes |
| Do the collected data allow to address the research questions? | Yes | No | Yes | Yes | Yes | Yes | Yes | Yes | No |
| Is the sampling strategy relevant to address the research question? | Yes | Yes | Yes | Yes | Yes | Yes | Yes | Yes | Yes |
| Is the sample representative of the target population? | Yes | No | Yes | Yes | No | No | Yes | No | No |
| Are the measurements appropriate? | Yes | No | Yes | Yes | Yes | Yes | Yes | Yes | No |
| Is the risk of nonresponse bias low? | No | Yes | No | Yes | No | No | No | Yes | No |
| Is the statistical analysis appropriate to answer the research question? | Yes | No | Yes | Yes | Yes | Yes | Yes | Yes | No |

| **Mixed Methods Appraisal Tool (MMAT), version 2018** | | |
| --- | --- | --- |
| **Category of study design** | **Mixed methods studies** | |
| **Author(s) of Corresponding Study(s)** | **Crombie, et al. (2024)** | **Leung, et al. (2020)** |
| Are there clear research questions? | Yes | Yes |
| Do the collected data allow to address the research questions? | Yes | Yes |
| Is there an adequate rationale for using a mixed methods design to address the research question? | Yes | Yes |
| Are the different components of the study effectively integrated to answer the research question? | Yes | Yes |
| Are the outputs of the integration of qualitative and quantitative components adequately interpreted? | Yes | Yes |
| Are divergences and inconsistencies between quantitative and qualitative results adequately addressed? | N/A | N/A |
| Do the different components of the study adhere to the quality criteria of each tradition of the methods involved? | Yes | No |
